# Postmortem CT is more accurate than clinical diagnosis for identifying the immediate cause of death in hospitalized patients: a prospective autopsy-based study

**DOI:** 10.1007/s00428-016-1937-6

**Published:** 2016-04-16

**Authors:** Kunihiro Inai, Sakon Noriki, Kazuyuki Kinoshita, Toyohiko Sakai, Hirohiko Kimura, Akihiko Nishijima, Hiromichi Iwasaki, Hironobu Naiki

**Affiliations:** Division of Molecular Pathology, Department of Pathological Sciences, Faculty of Medical Sciences, University of Fukui, 23-3 Matsuoka-Shimoaizuki, Eiheiji, Fukui 910-1193 Japan; Division of Tumor Pathology, Department of Pathological Sciences, Faculty of Medical Sciences, University of Fukui, 23-3 Matsuoka-Shimoaizuki, Eiheiji, Fukui 910-1193 Japan; Division of Radiology, Department of Radiology and Laboratory Medicine, Faculty of Medical Sciences, University of Fukui, 23-3 Matsuoka-Shimoaizuki, Eiheiji, Fukui 910-1193 Japan; Autopsy Imaging Center, Faculty of Medical Sciences, University of Fukui, 23-3 Matsuoka-Shimoaizuki, Eiheiji, Fukui 910-1193 Japan; Division of Infection Control, University of Fukui Hospital, 23-3 Matsuoka-Shimoaizuki, Eiheiji, Fukui 910-1193 Japan

**Keywords:** Autopsy, Diagnostic accuracy, Hospitalized patient, Immediate cause of death, Postmortem CT

## Abstract

Despite 75 to 90 % physician accuracy in determining the underlying cause of death, precision of determination of the immediate cause of death is approximately 40 %. In contrast, two thirds of immediate causes of death in hospitalized patients are correctly diagnosed by postmortem computed tomography (CT). Postmortem CT might provide an alternative approach to verifying the immediate cause of death. To evaluate the effectiveness of postmortem CT as an alternative method to determine the immediate cause of death in hospitalized patients, an autopsy-based prospective study was performed. Of 563 deaths from September 2011 to August 2013, 50 consecutive cadavers undergoing hospital autopsies with consent for additional postmortem CT at the University of Fukui were enrolled. The accuracy of determination of the immediate cause of death by postmortem CT was evaluated in these patients. Diagnostic discrepancy was also compared between radiologists and attending physicians. The immediate cause of death was correctly diagnosed in 37 of 50 subjects using postmortem CT (74 %), concerning 29 cases of respiratory failure, 4 of hemorrhage, 3 of liver failure and 1 of septic shock. Six cases of organ failure involving 13 patients were not identified as the cause of death by postmortem CT. Regarding the immediate cause of death, accuracy of clinical diagnosis was significantly lower than that of postmortem CT (46 vs 74 %, *P* < 0.01). Postmortem CT may be more useful than clinical diagnosis for identifying the immediate cause of death in hospitalized patients not undergoing autopsy.

**Electronic supplementary material:**

The online version of this article (doi:10.1007/s00428-016-1937-6) contains supplementary material, which is available to authorized users.

## Introduction

A critical decline in autopsy rates worldwide has occurred since the 1980s, with rates reaching less than 10 % of deaths in many countries [1, 2]. Many physicians are concerned about the decrease in both forensic and hospital autopsy rates, which is assumed to be partially due to the risk of infection, high costs, religious reasons, and clinical disinterest [3–7]. In contrast to the decrease in autopsy rates, the frequency of postmortem computed tomography (CT) imaging has increased as a supplement to autopsy in the past decade [5, 8]. In particular, postmortem CT in the forensic medical field has become a common analytical tool for examining cause of death, bone fracture, bleeding, anthropometry, and person identification [5, 9–16].

Ever since Virchow’s era, hospital autopsy, comprising macroscopic and microscopic investigations of tissues to elucidate the pathogenesis of the conditions leading to death, has provided physicians with epidemiological data, quality control of patient care, possibilities to re-evaluate diagnostic and therapeutic management of individual patients, and resources for medical education [1, 4, 6, 17]. In defining causes of death, three distinct levels can be distinguished: the underlying cause of death (primary underlying disease that initiates the events leading to death), the intermediate cause of death (critical conditions or comorbidities contributing to death), and the immediate cause of death (final disease or condition resulting in death) [18, 19]. The World Health Organization (WHO) lists underlying causes of death for each country according to the ICD-10 classification [20], and accurate vital statistics are utilized for cross-national surveillance of disease and hygiene. Analyses of the intermediate and immediate causes of death also provide practical information about the pathogenesis and critical events during the evolution of disease, which lead to a better understanding of disease and ultimately quality improvement in patient care. The accuracy of the clinical diagnosis regarding the underlying cause of death is estimated at 75 to 90 %, based upon several hospital autopsy validation studies [2, 4, 6, 21]. However, some studies have noted that the accuracy of clinical diagnosis of the immediate cause of death is lower than that of the primary cause of death, based upon hospital autopsy data [22–24]. Recent literature indicates that the correspondence rate of the immediate cause of death as determined by postmortem CT and hospital autopsy reaches approximately 70 % [25, 26], indicating that the accuracy of the determination of the immediate cause of death by postmortem CT is superior to that by clinical diagnosis.

These findings led us to hypothesize that postmortem CT might provide an alternative approach to verifying immediate cause of death. Accurate determination of cause of death is expected to improve understanding of disease progression, prognosis, and contribute to medical safety, even when a hospital autopsy is not performed. Therefore, the primary purposes of the present study were to compare immediate causes of death as determined by postmortem CT and by hospital autopsy and to identify strengths and weaknesses of postmortem CT. In addition, as a secondary endpoint, we compared the accuracy of determination of the immediate cause of death by postmortem CT with that of clinical diagnosis. We show that the accuracy of determination of the immediate cause of death by postmortem CT reached 70 %, which was significantly higher than that of clinical diagnosis.

## Methods

### Patient eligibility and profiles

From September 2011 to August 2013, 63 autopsies of 561 deaths at the University of Fukui Hospital were prospectively enrolled in this study. Fifteen deceased patients were excluded due to one of the following: denial of informed consent to participate in postmortem CT inspection (*n* = 1), weekend autopsy when only 1 pathologist was on duty (*n* = 9), and unsuitability for partial brain autopsy (*n* = 5). Two deceased subjects from other facilities, whose families agreed to allow their relative to join this study, were also included which makes for a study cohort of 50 deceased patients. Written informed consent was obtained from the family of each deceased patient prior to enrollment. All research protocols were approved by the ethics review board of our institute and conformed to the provisions of the Declaration of Helsinki.

Patient profiles are shown in Table 1. Of the 50 deceased patients (30 men and 20 women), 38 patients had a malignancy (27 solid tumors and 11 hematological malignancies), and the remaining 12 patients had a benign disease. The age range was 1 to 94 years (mean 67.3 ± 16.3 years; men, 66.7 ± 15.3 years; women, 68.3 ± 18.0 years, respectively). Main clinical diagnoses for hospitalizations and immediate causes of death decided by primary physicians were obtained from clinical records and application forms for hospital autopsy.

**Table 1:** Patient profiles

	Men	Women
Total number	30	20
Malignant	21	17
Solid malignancy	14	13
Hematological malignancy	7	4
Benign diseases	9	3
Age		
Mean	66.7 ± 15.3	68.3 ± 18.0
Median	69	68

### Postmortem CT imaging and diagnosis

Postmortem CT was performed at the autopsy imaging (Ai) center of the University of Fukui with an eight-slice multidetector CT scanner (Hitachi Medico, Tokyo, Japan) used exclusively for autopsies as described previously [7]. Each body was dressed in hospital pajamas, wrapped in a clear cotton sheet, and placed on a scanning table covered with waterproof paper. Inserted devices, such as endotracheal tubes and central venous catheters, were not removed until autopsy. The corpse was placed in supine position, and a full-body scan from the vertex to the toes was performed. The scanning conditions were 120 kV, 250 mA, 8 × 2.5 collimation, 1.125 pitch, 0.8-s rotation time, 5-mm slice thickness, and 5-mm increments [7, 27]. If necessary, images of 1-mm slice thickness were reconstructed by the radiology technician. No contrast reagent was used in this study. Mean imaging time was 15.5 ± 2.3 min.

The diagnostic procedure for determining the immediate cause of death used by radiologists is outlined in the flowchart in Supplemental Fig. 1. The CT images were interpreted by board-certified radiologists guided by clinical information, such as clinical records, laboratory data, and final antemortem CT imaging (FAMI), as modified previously [25]. Briefly, the radiologists analyzed the postmortem CT in a stepwise fashion. First, they checked the CT images for the presence or absence of a large hematoma/massive bleeding, and for patients with massive hemorrhage, the cause of death was established as secondary to hemorrhagic shock or cardiac tamponade. However, in addition to bleeding, cardiac tamponade can also be caused by congestive heart failure, renal failure, carcinomatous pericarditis, or hypoalbuminemia. Therefore, in case of cardiac tamponade by postmortem CT, the radiologist checked the CT value (Hounsfield unit) of the effusion because a hemorrhage shows a higher CT value than a pericardial effusion. Only cases of cardiac tamponade with a high CT value were diagnosed as hemorrhagic cardiac tamponade. Subsequently, other abnormalities were looked for in the CT images. When no abnormal findings were apparent, the immediate cause of death was described as “unknown.” When abnormalities were found in (an) organ(s), a tentative immediate cause of death was considered. When organ damage was confined to a single organ, the immediate cause of death was assumed to be associated with that organ, such as lung, liver, or kidney. In case of multi-organ abnormalities, the images allowed determination of the most predominantly injured organ. The validity of the tentative diagnosis was verified by comparing it with clinical information. When the tentative diagnosis conflicted with clinical information, the final radiological diagnosis was revised as “unknown.” On the other hand, in a few limited cases using clinical data, a more detailed diagnosis could be provided, including diagnosis such as acute respiratory distress syndrome (ARDS), pneumonia, or cancer-related death. When postmortem CT was compatible with ARDS, further evaluation was undertaken of a potential association with sepsis as follows: if a patient had been clinically diagnosed with sepsis/systemic inflammatory response syndrome (SIRS) or in case of vital signs of shock with identification of pre-existent infection sites such as pneumonia or abscess, the immediate cause of death was established as sepsis/septic shock.

**Fig. 1:**
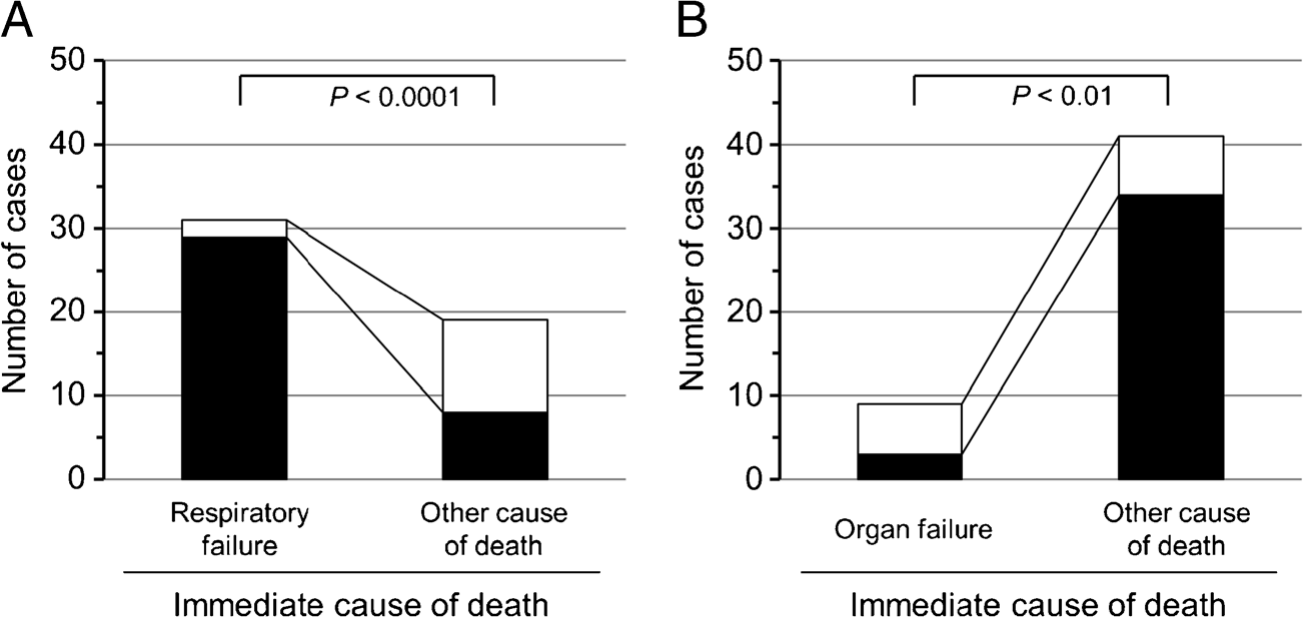
Preferentially diagnosed immediate cause of death by postmortem computed tomography. Accuracy between hospital autopsy (*open bar*) and postmortem computed tomography (*closed bar*) of respiratory failure (**a**) and organ failure (**b**) was statistically analyzed. Organ failures involved multi-organ failure, liver failure, and hepatorenal failure

### Hospital autopsy

Following postmortem CT, hospital autopsy was started within 15 min in the adjacent autopsy room in the Ai center. Hospital autopsies were usually performed within 24 h of the time of death. More specifically, when approval of autopsy and postmortem CT had been obtained during daytime (0830 to 1715 hours), postmortem inspection was completed on the same day. When approval had been obtained in the late evening, the cadaver was preserved overnight at 4 °C in supine position, and postmortem CT and autopsy were performed the next morning. Typical time until autopsy was 8.1 ± 7.6 h (median 5.1 h; range 1.25 to 41 h). All autopsies were started within 18 h of patient death, except for that of a 14-year-old female who died suddenly and for whom pathological inspection was required by the next of kin just before cremation.

Two thirds of the hospital autopsies were performed by pathology residents supervised by board-certified pathologists, and the remaining cases were performed by certified pathologists. Histological diagnoses were determined by at least two independent pathologists. They then made a flowchart of the likely pathogenesis, including clinical information and histological diagnoses, and determined the most critical element in progression of the disease (Supplemental Fig. 2). If more than one condition, such as respiratory failure and liver failure, were found in a patient, these were weighted according to the severity of clinical symptoms and laboratory data. Subsequently, sequential causes of death, including the underlying cause of death, intermediate cause of death, and immediate cause of death, were determined by certified pathologists. Concordance of the immediate cause of death in each case was finally reviewed by comparing causes determined by postmortem CT with those from clinical autopsy at the Ai meeting.

**Fig. 2:**
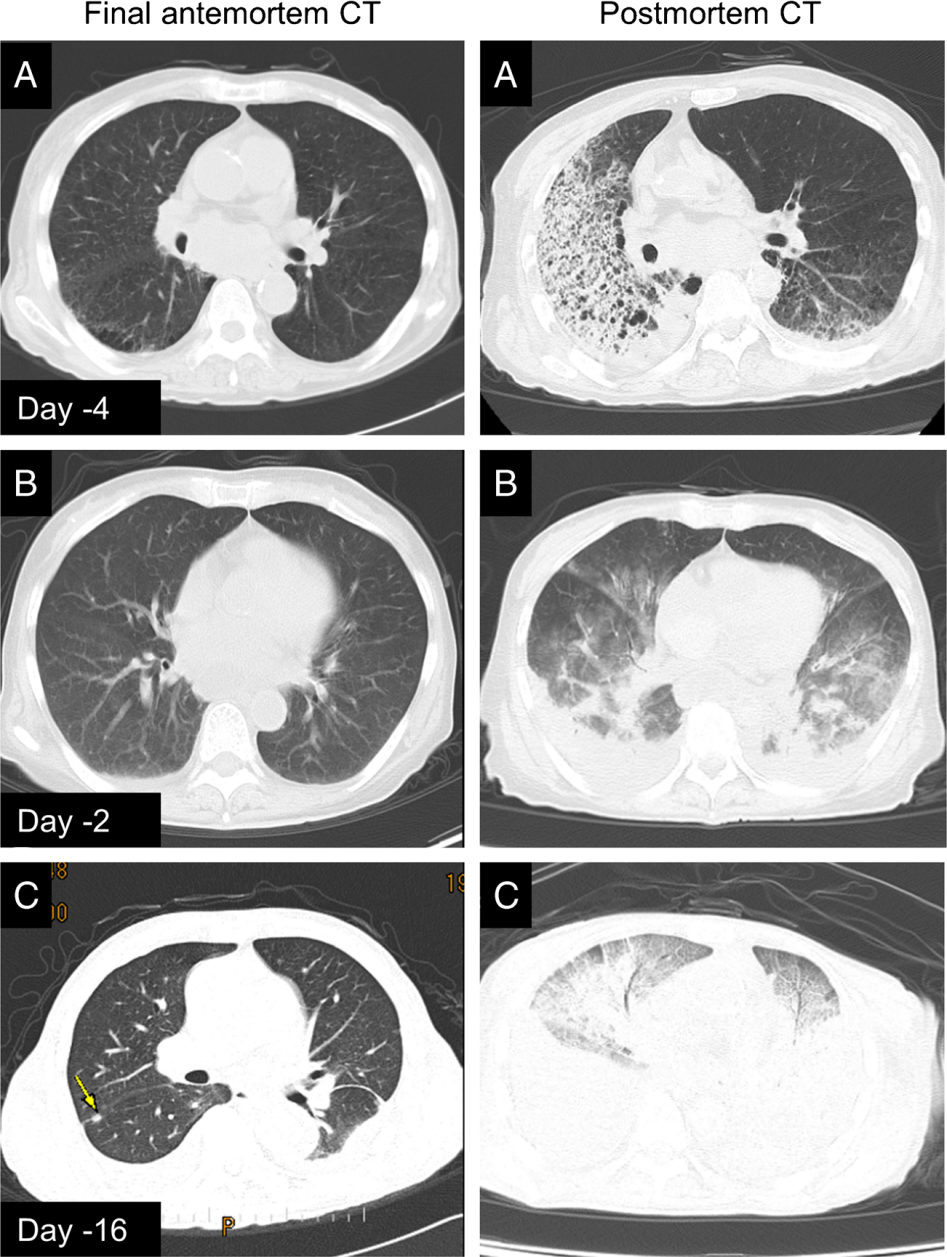
Three representative sets of final antemortem CT and postmortem CT images having dramatic changes in the lung fields. Case *A* represents bronchopneumonia, predominantly infiltrated in the right lung fields for 4 days. Case *B* shows acute respiratory distress syndrome (ARDS) with bilateral pleural effusion documented for only 2 days at the agonal phase in a patient with acute leukemia. Case *C* shows a patient with a solid malignancy who was considered by the attending physicians to have died due to cancer progression while the immediate cause of death was diagnosed as ARDS by postmortem CT. Yellow arrow: metastatic tumor. *CT* computed tomography

### Statistical analysis

All statistical analyses were performed with Ekuseru-Toukei 2010 software (Social Survey Research Information Co, Ltd., Tokyo, Japan). Statistical analysis was performed using the *χ*^2^ test. Values of *P* < 0.05 were accepted as statistically significant.

## Results

### Concordance between immediate cause of death determined by postmortem CT and by hospital autopsy

We first compared the immediate cause of death derived from postmortem CT with that determined by hospital autopsy, in order to evaluate diagnostic accuracy. The sequential causes of death, including underlying, intermediate, and immediate causes of death by autopsy, are listed in Supplemental Table 1. According to the level of concordance, cases were classified into four categories as follows: group 1 with complete concordance in terms of immediate cause of death between hospital autopsy, postmortem CT, and clinical diagnosis (19 patients); group 2 with concordance between autopsy and postmortem CT (18 patients); group 3 with concordance between autopsy and clinical diagnosis (4); and group 4 with any discordance between the three different diagnoses (9 patients, group 4) as shown in Supplemental Table 2. Concordance in terms of immediate cause of death between hospital autopsy and postmortem CT was 74 % (37/50 cases).

**Table 2:** Concordance of diagnostic accuracy of postmortem CT with hospital autopsy on immediate cause of death

Immediate cause of death by hospital autopsy	Immediate cause of death by postmortem CT		
	Concordance	Discordance	Unknown
Liver failure	3	4	
Multi-organ failure		1	
Hepatorenal failure		1	
Circulatory failure		2	
Acute myocardial infarction			1
Cardiac tamponade	2		
Hemorrhagic shock	2		
Septic shock	1		1
Pulmonary tumor embolism			1
Respiratory failure	29	2	
ARDS/DAD/sepsis	10		
Pneumonia/pulmonary abscess	9		
Interstitial pneumonia	2		
Airway obstruction/suffocation	2		
Passive atelectasis	1		
Pleuritis carcinomatosa	1		
Other respiratory failure	4		

*ARDS* acute respiratory distress syndrome, *CT* computed tomography, *DAD* diffuse alveolar damage

### Respiratory failure is the most frequent immediate cause of death in hospitalized patients by postmortem CT

**Fig. 3:**
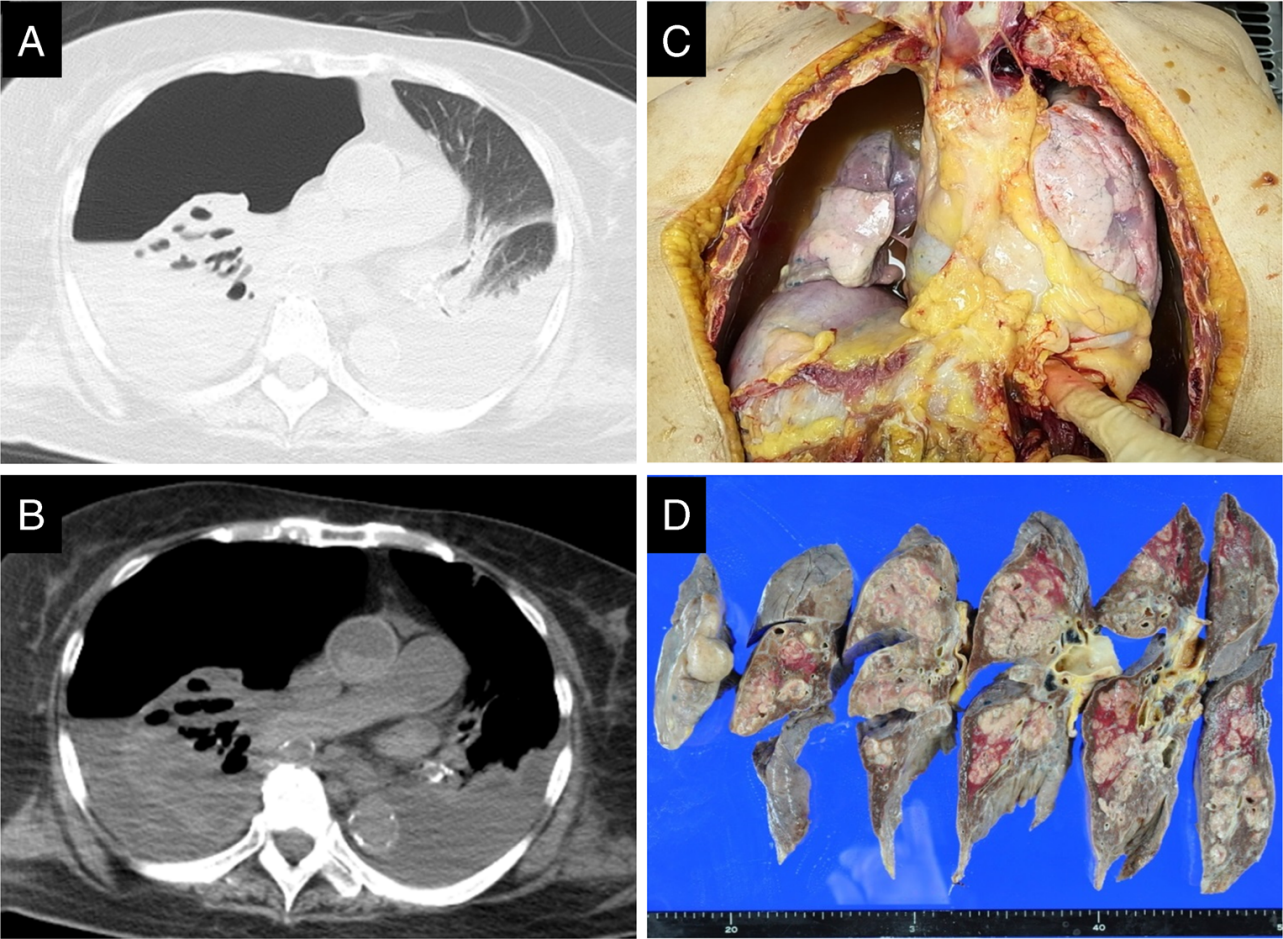
Vanishing pneumothorax by hospital autopsy. Pulmonary window setting (**a**) and mediastinal window setting (**b**) demonstrate a pneumothorax in the right lung that was easily detectable by postmortem computed tomography, while the lesion had disappeared during open-chest autopsy (**c**). The pneumothorax was induced by multiple lung metastases that were only diagnosed by hospital autopsy (**d**)

We then investigated the immediate cause of death most frequently diagnosed by postmortem CT. Concordance between hospital autopsy and postmortem CT is summarized in Table 2. Respiratory failure was the immediate cause of death in 29 cases, hemorrhage in 4 (2 cases of cardiac tamponade and 2 of hemorrhagic shock), liver failure in 3, and septic shock in 1 case. For these patients, the cause of death determined by hospital autopsy and postmortem CT was concordant. Respiratory failure was the most frequently diagnosed immediate cause of death, and the concordance for this diagnosis was significantly higher than that for other diseases (29/31 vs 8/19, *P* < 0.0001, Fig. 1a). Amongst the 29 cases of respiratory failure, 10 showed characteristics of ARDS/diffuse alveolar damage (DAD)/sepsis followed by 9 of pneumonia/pulmonary abscess, 2 of interstitial pneumonia, 2 of airway obstruction, 1 of passive atelectasis, and 1 of carcinomatous pleuritis (Table 2). Using postmortem CT and FAMI, these cases of respiratory failure were correctly diagnosed. Figure 2 shows three representative sets of pulmonary FAMIs and postmortem lung CTs useful for elucidating the cause of death. Surprisingly, postmortem CT often showed rapid changes in the agonal phase, lethal bronchopneumonia, and ARDS having developed in 4 and 2 days, respectively (Figs. 2a, 2b). In a fatal case after palliative treatment for multiple metastases (Fig. 2c), based upon FAMI and postmortem CT images, the immediate cause of death was established as respiratory failure due to bilateral massive pleural effusion and pleural opacities (ARDS), although the attending surgeons were convinced that cancer was the immediate cause of death until they obtained postmortem CT and autopsy results. In addition to clarifying such discrepancies, postmortem CT and hospital autopsy frequently provided complementary information. As an example, pneumothorax is more easily detectable by postmortem CT than by medical autopsy [2], as it (Figs. 3a, 3b) disappears with open-chest autopsy (Fig. 3c), while only at autopsy, the existence of multiple lung metastases could be established (Fig. 3d).

A cause of death could not be determined by postmortem CT for 13 of our 50 patients. These included 6 cases of organ failure, 4 of liver failure, 1 of hepatorenal failure, and 1 of multi-organ failure. Organ failure was the case in 6 of the 13 patients (46 %), followed by 2 cases of circulatory failure and 2 of respiratory failure (Table 2). Postmortem CT supported by laboratory test data could confirm 3 cases of liver failure. Organ failure diagnosis by postmortem CT lacked in accuracy (diagnostic accuracy: 3/9 vs 34/41, *P* < 0.01, Fig. 1b). As reported in previous publications [2, 5, 25], acute myocardial failure and pulmonary embolism are also diseases in which it is difficult to determine the immediate cause of death by postmortem CT.

### Immediate cause of death by postmortem CT is superior to clinical diagnosis

**Fig. 4:**
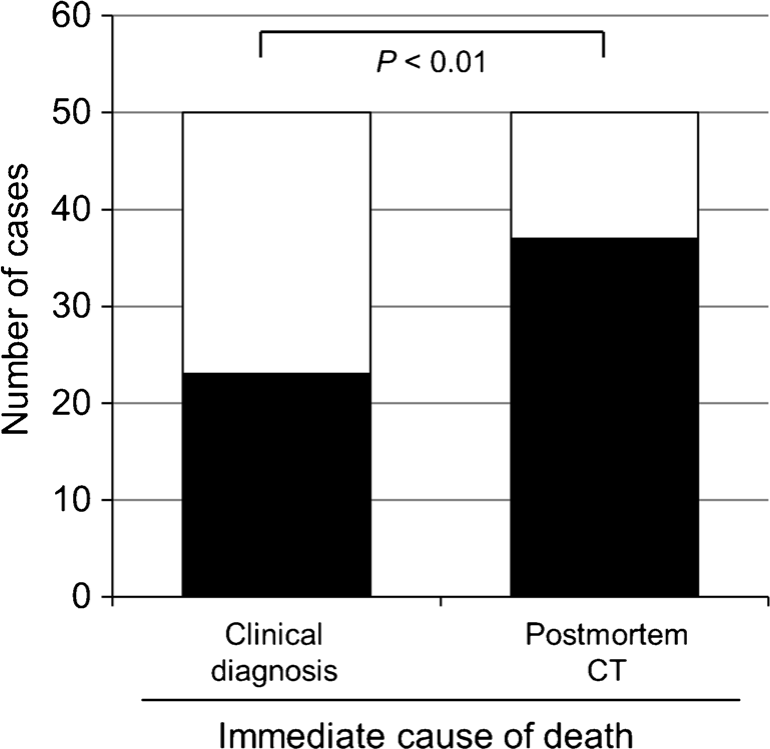
The diagnostic accuracy of immediate causes of death in clinical diagnoses by attending physicians versus postmortem computed tomography by radiologists. The accuracy of the determination of immediate cause of death between clinical diagnosis and postmortem CT was analyzed. The number of patients correctly diagnosed (*closed bar*) by each method was compared with those diagnosed by hospital autopsy (*open bar*). *CT* computed tomography

Several studies have evaluated the concordance between the clinical diagnosis of primary underlying diseases and what was found at hospital autopsy, with discrepancies in approximately 10 to 25 % of cases [2, 4, 6, 21]. Our data show a similar level of discordance (8/50, 16 %). In contrast, a few publications compare for the immediate cause of death the concordance between postmortem CT and clinical diagnosis. When we compared immediate cause of death determined by clinical diagnosis with that determined by postmortem CT, clinical diagnosis was concordant in 46 % (23 in 50 cases) compared to 74 % (37 in 50 cases) for postmortem CT (*P* < 0.01, Fig. 4).

The preferred clinical primary cause of death in cases of advanced cancer, when patients chose to receive best supportive care, supportive care, and/or palliative care [28] was cancer, which explains 13 of 27 misdiagnoses of immediate cause of death.

## Discussion

Recent advances in postmortem CT have improved the performance of radiology in diagnosing cause of death, which has accelerated its use in forensic medicine. Several publications have reported that postmortem CT can identify the immediate cause of death in about one third of deceased subjects [29, 30]. However, few reports have explored how well postmortem CT performs in cases of in-hospital death. We investigated the immediate cause of death comparing postmortem CT with clinical autopsy and found that 70 % of CT diagnoses correspond to those derived from autopsy. We assume that this discrepancy between forensic and hospital autopsy can be attributed to the availability of antemortem clinical information, such as clinical records, laboratory findings, and FAMI. In particular, dramatic changes between postmortem CT and FAMI were not uncommon in the agonal phase, as we have shown. In addition, our radiologists had access to medical data, including bacterial cultures, hemostatic data for sepsis and disseminated intravascular coagulation, and hepatorenal function tests, and decided on the immediate cause of death taking into account these clinical results. Similar concordance rates have been reported by virtual autopsy using CT of patients after in-hospital death, in which radiologists had been given antemortem clinical information for interpretation of postmortem CT images [25, 26]. These findings suggest that establishing the immediate cause of death in hospital patients is one of the most suitable applications of postmortem CT with an accuracy of approximately 70 %.

Increasing evidence has emerged that postmortem CT often fails to identify acute myocardial infarction, pulmonary embolism, and organ failure [2, 5, 25]. Our results confirm these limitations of postmortem CT. We also found for cases of organ failure, including multi-organ failure, liver failure, and hepatorenal failure significantly lower accuracy than for other causes of death by postmortem CT, although only for a limited number of cases antemortem clinical information could be used. For postmortem CT, only assessment of specific death-relevant organ dysfunction is difficult, even in patients with multiple organ metastatic tumors, because conventional postmortem CT is not performed using contrast enhancement. Hospital postmortem CT has been shown to be quite useful in the diagnosis of respiratory failure due to ARDS/DAD/septic shock, pneumonia, or pulmonary congestion. More than 90 % of the deceased patients were correctly diagnosed by postmortem CT, although reportedly, pneumonia is commonly misdiagnosed in coroners postmortem CT [5], which might be due to the time lapse between death and postmortem CT. We performed postmortem CT at 8.1 ± 7.6 h (median 5.1 h; range 1.25 to 41 h), whereas previous reports mention longer intervals up to 50 h [5, 25, 26]. Of note, the development of advanced opacity and consolidation shadows similar to those of pulmonary congestion and edema has been reported during the first few hours of the postmortem period in animal and human lungs [31, 32]. In addition, accumulation over time of pleural fluid has been found on postmortem CT [33]. We found clinical signs of respiratory failure to significantly correspond to hospital autopsy observations in this study. These findings suggest that in order to reliably establish respiratory failure by postmortem CT, it should be performed soon after death.

The past decade has shown a tendency to abandon aggressive treatment of patients with a poor prognosis, such as in the final stages of cancer, incurable neurological disorders, and cerebrovascular disease [28, 34–36]. Palliative care and best supportive care are expected to contribute to a reduction of healthcare cost by avoiding unnecessary treatment. In this context, however, it appears to become more difficult to discern between complications of the underlying disease and pathophysiologic mechanisms involved in terminal illness. As an example, as in the case in Fig. 3c, the clinicians were confident that the patient died from cancer progression, while both postmortem CT and hospital autopsy established ARDS/DAD as the immediate cause of death. In our case series, premortem clinical diagnosis of the underlying cause of death was correct in 84 % of cases (42/50, data not shown). This concordance rate was similar to that in previous reports [4, 6, 8, 21]. However, the accuracy of the clinical diagnosis of the immediate cause of death was significantly lower than that of the underlying cause of death (23/50 vs 42/50, *P* < 0.001). The most common clinical misdiagnosis was death due to cancer. Cancer should in most cases not be listed as immediate cause of death, but rather an underlying cause of death. Cancer as such might be the direct cause of death in only 10 % of cases [22, 37].

The accuracy of the immediate cause of death as established by postmortem CT was statistically significantly higher than that established by clinical diagnosis. Increased use of postmortem CT would lead to more reliable establishment of immediate causes of death, surpassed only by autopsy. Postmortem investigations such as autopsy and postmortem imaging are important, not only for elucidating pathogenesis of disease but also for educating residents and medical students [1, 38, 39]. Additionally, better insight in immediate and intermediate causes of death might provide improved guidelines for treatment [19].

The cost of postmortem CT has been estimated at approximately $500 by the Ministry of Health, Labour and Welfare, Japan, and that of clinical autopsy at $2100 by the Japanese Society of Pathology. Therefore, two FAMI CT examinations with postmortem CT still only cost half as much as conventional autopsy [40] or a minimally invasive autopsy with postmortem CT [41]. These findings suggest that wider use of postmortem CT should be considered in patients who die in the hospital, whether or not hospital autopsy is performed.

This study has some limitations. One limitation is the small number of cases studied. Second, many patients had a hematological disease, which may have increased the frequency of severe infection-related conditions such as ARDS, sepsis, and pneumonia. However, our recent autopsy-based study of a hematological condition [42] indicates that the incidence of this category of diseases is equal to that shown in the German, US, and Japanese populations [42–44], suggesting that our study population was not seriously biased.

In conclusion, we found that postmortem CT could identify the immediate cause of death in 70 % of in-hospital deaths confirmed by hospital autopsy. In addition, postmortem CT in combination with clinical information accurately diagnosed respiratory failure. Although our findings need to be confirmed, they indicate that postmortem CT contributes to more reliable diagnosis of immediate cause of death in hospital deaths.

## Electronic supplementary material

ESM 1 (PDF 24 kb)

ESM 2 (PDF 21 kb)

ESM 3 (PDF 69 kb)

ESM 4 (PDF 11 kb)
