# Supplementary material for: Postmortem CT is more accurate than clinical diagnosis for identifying the immediate cause of death in hospitalized patients: a prospective autopsy-based study
Source: Virchows Arch. 2016 Apr 16;469:101–9. doi: 10.1007/s00428-016-1937-6 (PMC4923108; doi:10.1007/s00428-016-1937-6)
Supplement: Supplementary file 1 — (PDF 24 kb) [file 428_2016_1937_MOESM1_ESM.pdf]

Supplemental Table 1. Sequential causes of death in each patient as diagnosed by pathologists

Group	Age	Sex	Time after death (h)	Underlying cause of death	Intermediate cause of death	Immediate cause of death
Group 1	82	M	1.25	Aspiration pneumonia	Diffuse alveolar damage	Respiratory failure due to diffuse alveolar damage
	89	F	1.75	Overt leukemia from myelodysplastic syndrome	Pneumonia with shock	Respiratory failure due to diffuse alveolar damage
	1	M	3.25	Primary immunodeficiency with thymic aplasia	Pneumonia	Diffuse alveolar damage
	81	F	14	Miliary tuberculosis	Diffuse alveolar damage	Diffuse alveolar damage
	88	F	15	Ascending colon cancer	Perforation of colon cancer	Septic shock due to pan-peritonitis
	75	M	2	Systemic lupus erythematosus	Invasive aspergillosis due to immunodeficiency	Respiratory failure due to septic shock
	69	F	9.5	Chronic myelomonocytic leukemia	Immunodeficiency	Respiratory failure due to diffuse alveolar damage
	63	M	10.75	Lingual cancer	Aspiration due to pharyngocutaneous fistula	Respiratory failure due to bronchopneumonia and pleural effusion
	68	M	27	Lung cancer	Multiple cancer metastases	Respiratory failure due to bronchopneumonia and pleural effusion
	78	M	2.5	Esophageal cancer	Aspiration pneumonia	Respiratory failure due to aspiration pneumonia
	64	F	2	Pancreatic cancer	Adverse effects of gemcitabine	Respiratory failure due to idiopathic interstitial pneumonia
	72	M	2.25	Usual interstitial pneumonia	Aspiration pneumonia + pulmonary bleeding	Respiratory failure due to CO_2 narcosis
	77	M	16	Liver cancer	Pleuritis carcinomatosa	Respiratory failure due to pleuritis carcinomatosa
	74	M	11	Traffic injury with polycythemia vera	Pulmonary thromboembolism	Respiratory failure due to pulmonary embolism
	51	M	7.25	Pancreatic head cancer	Pleuritis carcinomatosa with pneumothorax	Respiratory failure
	14	F	41	Unknown (Sudden death)	Diffuse alveolar bleeding	Suffocation
	45	F	3.5	Leiomyosarcoma of uterus	Peritonitis carcinomatosa	Hemorrhagic shock due to tumor necrosis
	60	F	1.75	Pancreatic head cancer	Ruptured metastatic liver tumor	Hemorrhagic shock due to perforation of liver tumor
	85	F	11	Lower bile duct cancer	Liver metastasis and obstructive jaundice	Liver failure
Group 2	65	M	5.5	Chronic myelomonocytic leukemia	Systemic inflammatory response syndrome	Respiratory failure due to septic shock
	62	F	4	Pancreatic cancer	Diffuse alveolar damage and lung metastasis	Respiratory failure due to diffuse alveolar damage
	79	M	15	Hodgkin lymphoma	Systemic inflammatory response syndrome	Diffuse alveolar damage
	73	M	6.25	Pancreatic head cancer	Disseminated intravascular coagulation	Diffuse alveolar damage
	62	M	1.75	Peritonitis carcinomatosa due to gastric cancer	Severe immunodeficiency	Respiratory failure due to bronchopneumonia and pleural effusion
	46	M	9.75	Amyotrophic lateral sclerosis	Aspiration	Respiratory failure due to pneumonia and pulmonary abscess
	76	F	2	Cecal cancer	Multiple lung abscesses	Respiratory failure due to pulmonary abscess
	86	M	21	Pancreatic head cancer	Bronchopneumonia + multiple lung metastases	Respiratory failure due to bronchopneumonia
	77	M	17	Pulmonary emphysema	Bronchopneumonia	Respiratory failure due to bronchopneumonia
	65	M	7	Drug-induced neutropenia	Severe systemic infection	Severe bronchopneumonia
	59	M	3	Diabetic nephropathy	Massive ascites	Respiratory failure due to passive atelectasis
	77	M	6.5	Ureteropelvic cancer	Carcinomatous lymphangitis + massive pleural effusion	Respiratory failure
	71	M	4.5	Usual interstitial pneumonia	Organizing pneumonia	Respiratory failure
	60	M	10	Mediastinal tumor	Tracheal compression by mass effect	Suffocation
	62	F	17.25	Angiosarcoma	Pericardial hemorrhage	Cardiac tamponade
	79	F	3.5	Gastric cancer	Pericarditis carcinomatosa	Cardiac tamponade
	71	M	3.5	Gastric body cancer	Metastatic liver cancer	Liver failure
	62	M	2.75	Pancreatic head cancer	Multiple liver metastasis	Liver failure
Group 3	68	M	1.75	Myelodysplastic syndrome	Immunodeficiency	Septic shock
	68	M	12.5	Old myocardial infarction	Acute coronary syndrome due to plaque rupture and thrombus formation	Acute myocardial infarction
	56	F	2	Endometrial cancer	Pan-peritonitis due to Pseudomonas infection	Multiple-organ failure
	71	M	14.75	Bile duct cancer	Sub-massive liver necrosis due to portal thrombus	Liver failure due to submassive liver necrosis
Group 4	57	M	2	Lymphoplasmacytic lymphoma/leukemia	Alveolar bleeding	Respiratory failure
	70	F	11	Carcinosarcoma of uterus	Bronchopneumonia	Respiratory failure
	67	M	9	Esophageal cancer	Multiple cancer metastases	Pulmonary tumor embolism
	76	M	2	Primary macroglobulinemia	Right heart failure	Suspect of acute circulatory failure
	94	F	3	Bronchopneumonia	Sepsis	Acute circulatory failure
	66	F	12.5	Liver cancer due to alcoholic liver cirrhosis	Radiation injury of the liver	Liver failure
	60	F	4.75	Gastric cancer	Liver metastasis	Liver failure
	81	F	3.5	Splenic marginal zone B-cell lymphoma	Tumor invasion of the cirrhotic liver	Liver failure
	64	F	1.75	Acute myelocytic leukemia	Tumor invasion of the liver	Hepatorenal failure
