# Supplementary material for: Postmortem CT is more accurate than clinical diagnosis for identifying the immediate cause of death in hospitalized patients: a prospective autopsy-based study
Source: Virchows Arch. 2016 Apr 16;469:101–9. doi: 10.1007/s00428-016-1937-6 (PMC4923108; doi:10.1007/s00428-016-1937-6)
Supplement: Supplementary file 2 — (PDF 21 kb) [file 428_2016_1937_MOESM2_ESM.pdf]

Supplemental Table 2. Concordance of the diagnosis of immediate cause of death by three different methods

Group	Age	Sex	Immediate cause of death		
			**Clinical diagnosis**	**Hospital autopsy**	**Postmortem CT**
Group 1	82	M	Acute respiratory distress syndrome	Respiratory failure due to diffuse alveolar damage	Respiratory failure due to acute respiratory distress syndrome
	89	F	Respiratory failure	Respiratory failure due to diffuse alveolar damage	Respiratory failure due to acute respiratory distress syndrome
	1	M	Respiratory failure	Diffuse alveolar damage	Respiratory failure due to acute respiratory distress syndrome
	81	F	Respiratory failure	Diffuse alveolar damage	Respiratory failure due to acute respiratory distress syndrome
	88	F	Pan-peritonitis	Septic shock due to pan-peritonitis	Septic shock
	75	M	Acute respiratory distress syndrome	Respiratory failure due to septic shock	Respiratory failure
	69	F	Sepsis due to febrile neutropenia	Respiratory failure due to diffuse alveolar damage	Respiratory failure due to pneumonia
	63	M	Respiratory failure due to pneumonia	Respiratory failure due to bronchopneumonia and pleural effusion	Respiratory failure due to pneumonia
	68	M	Respiratory failure	Respiratory failure due to bronchopneumonia and pleural effusion	Respiratory failure
	78	M	Pneumonia	Respiratory failure due to aspiration pneumonia	Respiratory failure
	64	F	Interstitial pneumonia	Respiratory failure due to idiopathic interstitial pneumonia	Respiratory failure due to interstitial pneumonia
	72	M	CO_2 narcosis	Respiratory failure due to CO_2 narcosis	Respiratory failure due to usual interstitial pneumonia
	77	M	Respiratory failure	Respiratory failure due to pleuritis carcinomatosa	Respiratory failure
	74	M	Pulmonary thromboembolism	Respiratory failure due to pulmonary embolism	Respiratory failure
	51	M	Respiratory failure	Respiratory failure	Respiratory failure
	14	F	Pulmonary hemorrhage	Suffocation	Respiratory failure
	45	F	Hemorrhagic shock due to gastrointestinal tract bleeding	Hemorrhagic shock due to tumor necrosis	Hemorrhagic shock
	60	F	Hemorrhagic shock due to ruptured metastatic liver tumor	Hemorrhagic shock due to perforation of liver tumor	Hemorrhagic shock
	85	F	Liver failure	Liver failure	Liver failure
Group 2	65	M	Acute leukemia	Respiratory failure due to septic shock	Respiratory failure due to sepsis and pneumonia
	62	F	Interstitial pneumonia	Respiratory failure due to diffuse alveolar damage	Respiratory failure due to acute respiratory distress syndrome
	79	M	Pneumonia	Diffuse alveolar damage	Respiratory failure due to acute respiratory distress syndrome
	73	M	Acute renal failure	Diffuse alveolar damage	Acute respiratory distress syndrome
	62	M	Peritonitis carcinomatosa	Respiratory failure due to bronchopneumonia and pleural effusion	Respiratory failure due to pneumonia
	46	M	Respiratory arrest	Respiratory failure due to pneumonia and pulmonary abscess	Respiratory failure due to pneumonia
	76	F	Cachexia	Respiratory failure due to pulmonary abscess	Respiratory failure due to pulmonary abscess
	86	M	Pancreatic head cancer	Respiratory failure due to bronchopneumonia	Respiratory failure
	77	M	Metabolic acidosis	Respiratory failure due to bronchopneumonia	Respiratory failure
	65	M	Respiratory failure	Severe bronchopneumonia	Respiratory failure due to acute respiratory distress syndrome
	59	M	Hemorrhagic shock due to gastrointestinal tract bleeding	Respiratory failure due to passive atelectasis	Respiratory failure due to acute respiratory distress syndrome
	77	M	Acute renal failure	Respiratory failure	Respiratory failure due to passive atelectasis
	71	M	Pulmonary hypertension	Respiratory failure	Respiratory failure
	60	M	"So-called" cancer death	Suffocation	Suffocation
	62	F	Congestive heart failure	Cardiac tamponade	Cardiac tamponade
	79	F	Acute renal failure due to peritonitis carcinomatosa	Cardiac tamponade	Respiratory and circulatory failure due to cardiac tamponade
	71	M	"So-called" cancer death due to metastatic liver cancer	Liver failure	Suspect of liver failure
	62	M	"So-called" cancer death	Liver failure	Suspect of liver failure
Group 3	68	M	Septic shock	Septic shock	Unknown
	68	M	Acute myocardial infarction	Acute myocardial infarction	Unknown
	56	F	Multi-organ failure	Multiple-organ failure	Pan-peritonitis
	71	M	Liver failure	Liver failure due to sub-massive liver necrosis	Liver abscess
Group 4	57	M	"So-called" cancer death	Respiratory failure	"So-called" cancer death
	70	F	"So-called" cancer death	Respiratory failure	"So-called" cancer death
	67	M	Esophageal cancer	Pulmonary tumor embolism	Unknown
	76	M	Unknown	Suspect of acute circulatory failure	Congestive heart failure with unknown reasons
	94	F	Sepsis	Acute circulatory failure	Suspect of respiratory failure
	66	F	Disseminated intravascular coagulation	Liver failure	Respiratory failure due to lung congestion
	60	F	Peritonitis carcinomatosa	Liver failure	Cachexia
	81	F	Acute renal failure due to tumor lysis syndrome	Liver failure	"So-called" cancer death
	64	F	Acute leukemia	Hepatorenal failure	Sepsis

CT, computed tomography
