# Supplementary material for: Postmortem CT is more accurate than clinical diagnosis for identifying the immediate cause of death in hospitalized patients: a prospective autopsy-based study
Source: Virchows Arch. 2016 Apr 16;469:101–9. doi: 10.1007/s00428-016-1937-6 (PMC4923108; doi:10.1007/s00428-016-1937-6)
Supplement: Supplementary file 3 — (PDF 69 kb) [file 428_2016_1937_MOESM3_ESM.pdf]

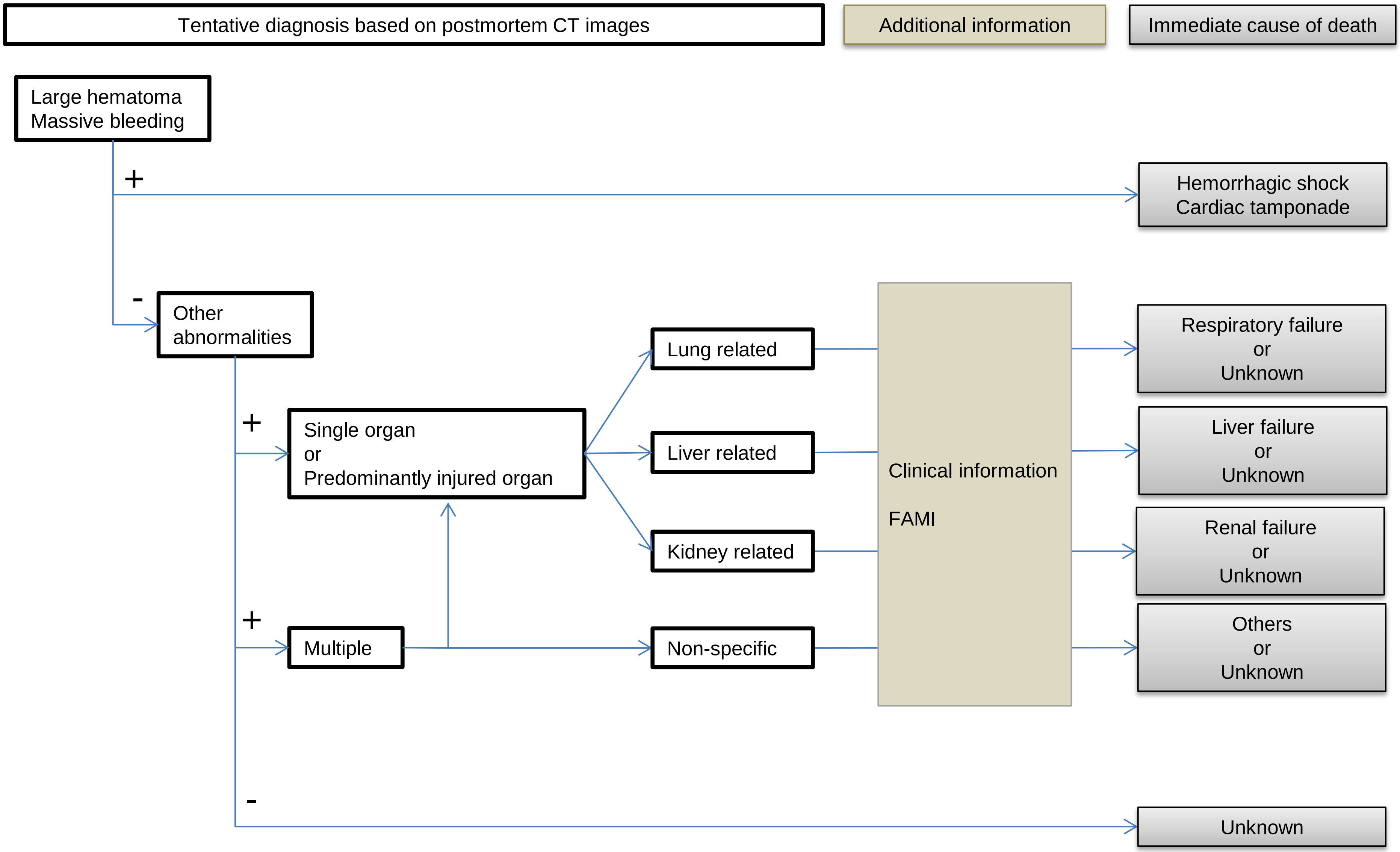

Supplemental Figure 1. Diagnostic procedure flowchart used by radiologists for determining immediate cause of death. The radiologists checked the existence of large hematoma or massive bleeding followed by the exploration of organ injuries for tentative diagnosis. Then, they verified the validity of the tentative diagnosis using antemortem clinical information such as laboratory data, vital signs, and FAMI, and finally determined the immediate cause of death. FAMI, final antemortem CT imaging
