# Supplementary material for: Postmortem CT is more accurate than clinical diagnosis for identifying the immediate cause of death in hospitalized patients: a prospective autopsy-based study
Source: Virchows Arch. 2016 Apr 16;469:101–9. doi: 10.1007/s00428-016-1937-6 (PMC4923108; doi:10.1007/s00428-016-1937-6)
Supplement: Supplementary file 4 — (PDF 11 kb) [file 428_2016_1937_MOESM4_ESM.pdf]

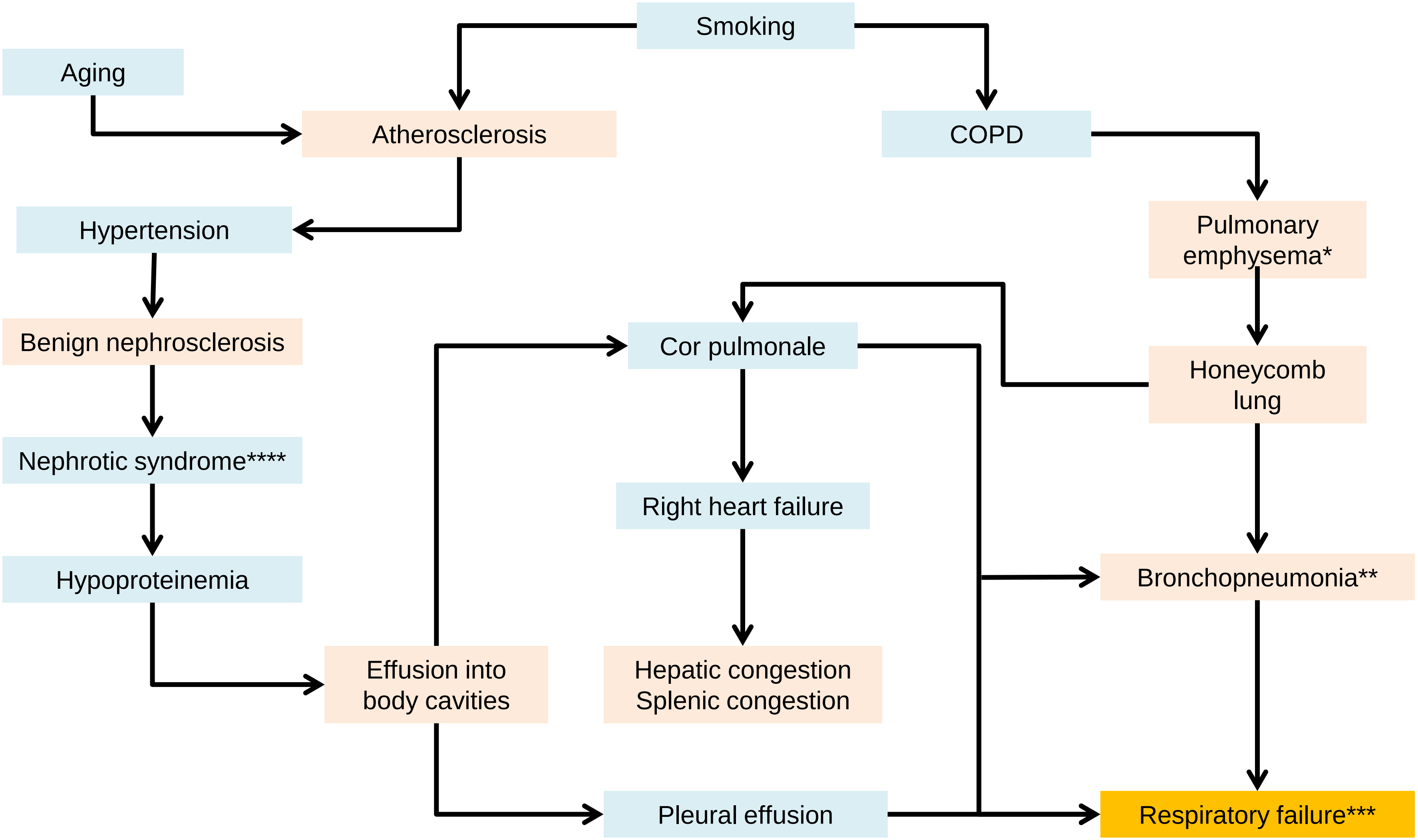

Supplemental Figure 2. Representative diagnostic procedure flowchart analyzed by pathologists for determining the causes of death (77-year-old male with pulmonary emphysema, Group 2).
The pathologists made a flowchart of the pathogenesis in each patient according to the antemortem clinical information (blue) and autopsy findings (orange). Then, they investigated the most critical procedure of the disease progression, and determined the underlying cause of death*, intermediate cause of death**, and immediate cause of death***. In this case, the immediate cause of death was clinically diagnosed as nephrotic syndrome****, however, the final cause of death was revised as respiratory failure due to bronchopneumonia by the pathologists.
COPD, chronic obstructive pulmonary disease
